# From Basics to Benchmarks: Evaluating Sample Adequacy, PD‐L1 Expression, and Molecular Profiling in Effusion Samples of Lung Adenocarcinoma

**DOI:** 10.1002/dc.70132

**Published:** 2026-04-26

**Authors:** Harpreet Virk, Claire Michael, Keri Ann Pfeil, Navid Sadri, Omid Savari, Aparna Harbhajanka

**Affiliations:** ^1^ Department of Pathology University Hospitals Cleveland Medical Center, Case Western Reserve University Cleveland Ohio USA; ^2^ Clinical Research Biostatistician, UH Center for Clinical Research Cleveland Ohio USA

**Keywords:** effusion cytology, lung adenocarcinoma, malignant pleural effusion, next generation sequencing, PD‐L1 expression, tumor cellularity

## Abstract

**Introduction:**

Lung adenocarcinoma commonly causes malignant pleural effusion (MPE), a condition with poor prognosis and limited treatment options. Pleural effusion specimens offer a minimally invasive source for diagnosis and molecular testing. This study evaluates cytologic adequacy for molecular analysis, characterizes mutational and PD‐L1 profiles, and correlates findings with survival outcomes.

**Methods:**

Over 10 years, a review of 2400 MPE identified 94 lung adenocarcinoma cases that underwent next generation sequencing (NGS). Patient demographics, cytological characteristics, molecular features, and survival outcome were integrated for analysis.

**Results:**

The mean effusion volume submitted for cytological and molecular testing was 589.8 mL (range 4–2790 mL) and mean tumor cellularity was 40% (range 10%–90%). NGS identified mutations in 86/94 cases (91%). TP53 was the most common alteration (42/94, 44.6%). FDA‐approved biomarkers were detected: KRAS (28/94, 29.7%); EGFR (15/94, 15.9%); BRAF (8/94, 8.5%); ALK (3/94, 4.2%), ROS1 (2/94, 2.1%), RET fusions (1/94, 1%); and MET amplification (2/94, 2.1%). Smoking was associated with bloody effusions (*p* = 0.049), frequent TP53 mutations (*p* = 0.018), and fewer EGFR mutations (*p* = 0.012). Patients with effusions as primary presentation was associated with higher mortality (*p* = 0.002), frequent TTF‐1 positivity (*p* = < 0.001) and frequent BRAF mutations (*p* = 0.049). TTF‐1 positivity was associated with higher PD‐L1 scores (*p* = 0.035). Survival was shorter in male patients (*p* = 0.045), smokers (*p* = 0.025), concurrent pericardial effusions (*p* = 0.027), and with TP53 mutation (*p* = 0.002).

**Conclusions:**

Molecular testing of effusion samples is feasible even at low fluid volumes, provided tumor cellularity is adequate. Prioritizing tumor fraction over volume ensures a higher likelihood of successful molecular analysis. Effusion‐based NGS reliably identifies actionable alterations with diagnostic and prognostic relevance. Male patients, smokers, concurrent or primary presentation with MPE, and those with TP53 mutations had poorer outcomes.

## 1 Introduction

Lung adenocarcinoma remains a major contributor to cancer‐related mortality worldwide and is one of the most frequent causes of MPE, a complication associated with poor prognosis and limited therapeutic options [1]. The identification of actionable molecular alterations has reshaped the treatment landscape of lung cancer, enabling the use of targeted therapies and immunotherapy to significantly improve clinical outcomes [2, 3]. Molecular profiling traditionally relies on core tissue biopsies, which can be difficult to obtain in patients presenting with advanced disease or medically fragile states. In these scenarios, pleural effusion, a common manifestation in metastatic lung adenocarcinoma, offers a minimally invasive and readily accessible source for both diagnostic evaluation and molecular testing. Recent advances in NGS have expanded the utility of cytology specimens, allowing comprehensive genomic analysis even in samples with limited tumor cellularity [4, 5]. Despite these technological improvements, real‐world data on the performance of NGS in pleural effusion samples remain relatively scarce. Moreover, the interplay between molecular alterations detected in MPE and the clinicopathologic features of the effusion is not fully understood. Additionally, clear guidelines on minimum fluid volume and tumor cellularity for adequate molecular testing remain limited and inconsistently defined. While characterizing the mutational landscape of MPE can aid in clinical triaging, it is equally important to explore cytological features associated with specific genetic alterations. Such insights could deepen our understanding of tumor behavior in the pleural space and refine how cytopathologists assess sample adequacy and interpret molecular test results [6, 7].

We performed targeted NGS on pleural effusion samples from lung adenocarcinoma patients to assess diagnostic yield, define genomic alterations, and evaluate associations with clinical, cytologic, and survival outcomes. Our study sought not only to map the molecular profile of MPE in lung adenocarcinoma but also to evaluate how these mutations relate to effusion characteristics, tumor markers, and patient prognosis. Ultimately, this integrated approach aims to enhance the clinical value of pleural effusion cytology as both a diagnostic and prognostic tool in advanced lung cancer.

### 1.1 Materials and Methods

This study was approved by the institutional review board of University Hospitals Cleveland Medical Center (UHCMC). A total of 2400 malignant effusion samples were retrospectively reviewed through natural language search in EPIC software from 2017 to 2025. Ninety‐Four (94) samples from Lung adenocarcinoma with pleural effusion were identified and subjected to tumor profiling by NGS using post‐ThinPrep PreservCyt (PTPC) fluid or cell block scrapings. ThinPrep and cell block slides were made for all cytology samples and were reviewed by experienced cytopathologists to confirm the diagnosis and summarize morphologic features as a part of routine patient care diagnostics. Immunohistochemistry (IHC) was performed following manufacturers' recommendations. IHC was performed on all 94 cases with positive cytology confirming metastatic adenocarcinoma from lung primary. Clinicopathologic parameters, including patient age, history of smoking and alcohol consumption, and effusion history (e.g., bilateral versus unilateral pleural effusion, associated pericardial effusions) were noted. Programmed death‐ligand 1 (PD‐L1) 22C3 by IHC was performed using Monoclonal Mouse Anti‐PD‐L1, Clone 22C3 on the Optiview detection on a Ventana BenchMark Ultra. This assay is indicated as an aid in identifying NSCLC patients for treatment with pembrolizumab (KEYTRUDA).

Follow‐up data including overall survival (OS) and progression‐free survival (PFS) were documented. Effusion fluid characteristics such as volume, gross appearance, and microscopic tumor cellularity were assessed through a review of clinical charts and pathology reports and were reviewed by expert cytopathologists. Molecular analysis by targeted NGS was performed with attention to clinical details and pre‐analytical tumor cellularity [tumor‐to‐non‐tumor (T:NT) cell ratio]. NGS was performed both in cell blocks and PTPC fluid. Mutations classified as variants of unknown significance (VUS) were also noted. The in‐house NGS panel detects targeted clinically relevant single nucleotide variants, insertion and deletions (< 30 bp), and whole gene high copy number amplifications in a select group of genes. Patients were followed from the time of lung cancer diagnosis and effusion development to the last encounter or death. Pearson's Chi‐squared test and fisher's exact test were used for categorical variables and Welch Two Sample *t*‐test for continuous variables. Time from lung cancer diagnosis to effusion was compared using Wilcoxon and Kruskal‐Wallis tests by cellularity, laterality, loculation, pericardial effusion, and fluid color. Univariate cox proportion hazard models were used to determine risk of death after diagnosis of lung cancer and effusion as separate models for tumor cellularity groups, unilateral versus bilateral pleural effusions, loculated effusions, associated pericardial effusions, and pleural fluid color. Estimates of hazard ratios (HR), and 95% confidence intervals (CI) were used to describe the hazard of each outcome. Additionally, a fishers exact test was used to determine any associations between cellularity and gene mutation type.

## 2 Results

### 2.1 Patient Demographics

NGS based molecular analysis was conducted on effusion samples from 94 patients diagnosed with lung adenocarcinoma. The cohort comprised 44 males and 50 females (M:F ratio 1:1.2); 74/94 (79%) were Caucasian, 19/94 (20%) were African American, and 1/94 (1%) patient was Asian. The median age at diagnosis was 74 years (range 43–100 years). A significant proportion of patients (74/94, 79%) reported a history of smoking, while 43% (40/94) had a documented history of alcohol consumption. All cases were confirmed as adenocarcinoma through a combination of cytological evaluation and IHC profiling. The majority (76/94, 81%) were TTF‐1 positive. The rest of the cases (18%, 19%) were TTF‐1 negative, and their lung origin was proved by a combination of morphology, additional immunostains (CK7, Napsin A), and molecular findings.

### 2.2 Effusion Characteristics

**FIGURE 1:**
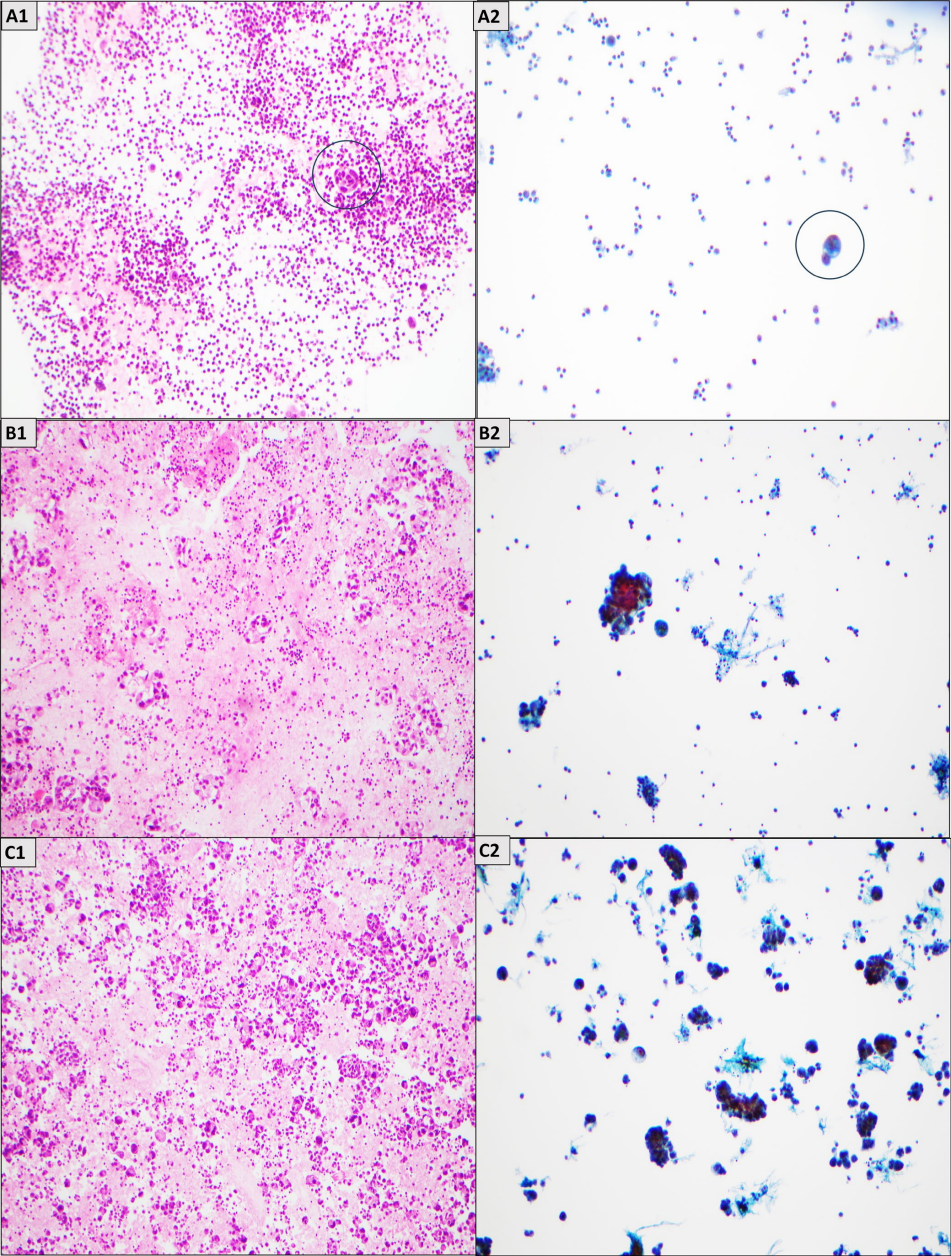
Representative pictures of Thin prep and corresponding cell blocks to demonstrate tumor cellularity/tumor: Non tumor ratio (T:NT) across three groups. A1 and A2: Tumor cellularity < 10%, scant tumor cells (circled) in the background of numerous inflammatory cells; B1 and B2: Tumor cellularity 11%–49%, moderately cellular sample admixed with few inflammatory cells and reactive mesothelial cells; C1 and C2: Tumor cellularity ≥ 50%, shows abundant tumor cell clusters. [Color figure can be viewed at wileyonlinelibrary.com]

All specimens analyzed were pleural fluid effusion samples (Table 1). Most patients (78/94, 83%) presented with unilateral effusions, while approximately half (48/94, 51%) exhibited loculated effusions. Loculated effusions are characterized by fluid collections that become confined within fibrous septations, rather than freely circulating within the pleural cavity, and are often clinically difficult to drain [8]. In our study, 87% (82/94) developed early effusions, defined as occurring within 1 year of the initial diagnosis. Of these, 63 patients (77%) presented with concurrent effusions, indicating that the effusion was present at the time of primary lung cancer diagnosis. The mean ± SD volume of fluid submitted for cytological and molecular testing was 589.8 ± 602.4 mL (range 4–2790 mL). Tumor cellularity was assessed on cell block preparations in 81 cases and was defined as the proportion of tumor cells relative to all other nucleated cells, including reactive mesothelial and inflammatory cells. The cell blocks of rest 13 cases could not be retrieved from the archives for assessment of cellularity. The mean tumor cellularity across the cohort was 40% (range, 10%–90%). Based on tumor content, cases were stratified into three groups (Figure 1): low cellularity (0%–10%, *n* = 5), intermediate cellularity (11%–49%, *n* = 38), and high cellularity (≥ 50%, *n* = 38). The mean submitted fluid volumes were 715 mL for samples with ≤ 10% cellularity, 772 mL for those with 11%–49% cellularity, and 532 mL for samples with ≥ 50% cellularity. We evaluated the relationship between submitted sample volume and tumor cellularity (Table 2). Notably, very low volume samples (≤ 10 mL) demonstrated tumor cellularity at least > 10%, with the majority (5/8, 63%) exhibiting high tumor cellularity (> 50%). Additionally, samples with still lower volumes of 11–50 mL also predominantly showed high tumor cellularity (> 50%; 6/9, 67%). In contrast, four of five samples with low tumor cellularity (< 10%) had fluid volumes of at least > 100 mL. Molecular analysis was successfully performed with sample volumes as low as 5 mL. This sample had T:NT ratio of 55%. No significant differences were noted between tumor cellularity groups and demographic features like age, sex, alcohol and smoking history. No significant difference was noted with tumor cellularity and timing of effusions (concurrent/early effusions versus late or median time to effusions). Those with smoking history had more common bloody effusions than non‐smokers (89.5% versus 10.5%, *p*: 0.049). TTF‐1 positivity was significantly more frequent in patients who presented with either concurrent effusions (93.5 versus 6.5% *p* = < 0.001), or those with early effusions within 1 year (85.2 versus 14.8% *p* = 0.040). No significant racial differences were noted in African American versus Caucasian race.

**TABLE 1:** Effusion characteristics in our cohort.

Characteristic	*N* = 94
Laterality	
Unilateral	78 (83%)
Bilateral	16 (17%)
Loculated effusions	
Yes	48 (51%)
No	46 (49%)
Concurrent pericardial effusion	
Yes	16 (17%)
No	78 (83%)
Concurrent effusions	
Yes	63 (67%)
No	31 (33%)
Bloody effusions^a^	
Yes	38 (40%)
No	56 (60%)
Mean volume of sample	589.8 mL (range 4–2790 mL)
Mean tumor cellularity	40% (range, 10%–90%)
TTF‐1 positivity	76/94 (81%)

^**a**^ Those with smoking history had more common bloody effusions than non‐smokers (89.5% versus 10.5%, *p*: 0.049).

**TABLE 2:** Effusion volume and tumor cellularity.

Volume of sample	Number of specimens with corresponding tumor cellularity (*n* = 81)		
	≤ 10%	11%–49%	≥ 50%
≤ 10 ml	0	3	5
11–50 ml	1	2	6
51–100 ml	0	3	7
100–500 mL	1	11	2
> 500 ml	3	19	18

### 2.3 Tumor Proportion Score and Effusion Characteristics

PD‐L1 protein expression was assessed using the Tumor Proportion Score (TPS), defined as the percentage of viable tumor cells exhibiting partial or complete membranous staining of any intensity. PD‐L1 22C3 by IHC with Interpretation is a qualitative immunohistochemical assay which was performed using Monoclonal Mouse Anti‐PD‐L1, Clone 22C3 intended for use in the detection of PD‐L1 protein in FFPE tissue using the Optiview detection on a Ventana BenchMark Ultra. The specimen submitted for testing should contain at least 100 viable tumor cells to be considered adequate for evaluation. PD‐L1 expression was categorized as high expression (TPS ≥ 50%), low expression (TPS 1%–49%), and no expression (TPS < 1%) [9]. TPS results were available for 76 of 94 patients (81%). PDL‐1 was not available in 18 cases due to insufficient cell block material. In these cases, PD‐L1 testing was performed on corresponding surgical resection or biopsy specimens as part of routine clinical care.

For those with available PDL1 testing on cytology cell blocks, 14 patients (18%) demonstrated no PD‐L1 expression, whereas 62 patients (82%) were PD‐L1–positive, including 28 (37%) with low expression and 34 (45%) with high expression. Loculated effusions were significantly more frequent among PD‐L1–positive cases (72.2% vs. 27.8%; *p* = 0.046). Additionally, TTF‐1 positivity was strongly associated with high PD‐L1 expression (97.1% vs. 2.9%; *p* = 0.035).

### 2.4 Molecular Landscape of Patients With Lung Adenocarcinoma and MPE

NGS analysis was successfully performed on all 94 pleural effusion samples, yielding detectable mutations in 86 cases (91%) and in 8 patients, no pathogenic mutations were detected. NGS was performed both in cell block (36%) and PTPC (64%). The most frequently observed alteration was TP53 mutation, detected in 42 cases (44.6%). Among these, point mutations constituted the majority (31/42, 74%), while the remainder comprised frameshift and splice site variants. FDA‐approved biomarkers were detected: KRAS (28/94, 29.7%); EGFR (15/94, 15.9%); BRAF (8/94, 8.5%); ALK (3/94, 4.2%), ROS1 (2/94, 2.1%), RET fusions (1/94, 1%); and MET amplification (2/94, 2.1%). Spectrums of other mutations are summarized in Table 3. FDA approved biomarkers are highlighted.

**TABLE 3:** Frequency of genetic mutations in pleural effusions in patients with lung adenocarcinoma in our study.

Mutation	Frequency
No mutation	8/94 (8.5%)
TP53 mutation	42/94 (44.6%)
KRAS mutation	28/94 (29.7%)
EGFR^a^ mutation	15/94 (15.9%)
BRAF^a^ mutation	8/94 (8.5%)
FGFR amplification	5/94 (5.3%)
KEAP1 mutation	5/94 (5.3%)
CDK 4/6 amplification	4/94 (4.2%)
ALK Fusion^a^	4/94 (4.2%)
MYC amplification	3/94 (3.1%)
CTNNB1 mutation	3/94 (3.1%)
CDKN2A mutation	3/94 (3.1%)
MAP2K1 mutation	2/94 (2.1%)
ERBB2 amplification	2/94 (2.1%)
MET amplification^a^	2/94 (2.1%)
FGFR mutation/fusion	2/94 (2.1%)
ERBB2 mutation	2/94 (2.1%)
ROS1 Fusions^a^	2/94 (2.1%)
PIK3CA mutation	2/94 (2.1%)
CCND1 mutation	2/94 (2.1%)
ESR1 mutation	1/94 (1%)
U2AF1 mutation	1/94 (1%)
IDH1 mutation	1/94 (1%)
NFE2L2 mutation	1/94 (1%)
FBXW7 mutation	1/94 (1%)
PTEN mutation	1/94 (1%)
SMAD4 mutation	1/94 (1%)
CREBBP mutation	1/94 (1%)
ALK mutation	1/94 (1%)
RET Fusions^a^	1/94 (1%)
PDGFRA mutation	1/94 (1%)
KIT amplification	1/94 (1%)
Rictor amplification	1/94 (1%)
JAK2V617F mutation	1/94 (1%)

^a^ FDA‐approved therapy biomarkers.

Patients who smoked were more likely to have TP53 mutation (51.4% versus 48.6% *p* = 0.018) and less frequently present with EGFR mutation (89.2% versus 10.8% *p* = 0.012). Patients with concurrent effusions more frequently harbored BRAF mutation in their effusion specimens (*p* = 0.049). TP53 was more common with unilateral effusions compared to bilateral effusions (50% versus 19% *p* = 0.022). EGFR mutation was significantly more common with > 10% tumor cellularity groups (80%) compared to those with less than 10% (20% *p* = 0.05). We did not find any difference in tumor cellularity and other mutations.

### 2.5 Molecular Analysis of Primary Tumor Versus Effusions

Paired molecular analysis of primary tumor specimens (lung biopsy or resection) and corresponding effusion samples was available for nine patients (Table 4). Four primary tumors showed no detectable mutations; however, two of these had clinically relevant mutations (TP53 and KRAS) on subsequent effusion testing, while the remaining two remained mutation‐negative. Concordant molecular profiles between primary tumor and effusion specimens were observed in four patients. In one case, additional mutations (ESR1, FBXW7, and PTEN) were identified in the effusion specimen, with a shared TP53 mutation present in both the primary tumor and effusion specimen.

**TABLE 4:** Molecular result comparison on primary tumor (lung Biopsy/surgical resection) for 9 patients compared to their corresponding effusion specimens.

S/no.	Molecular results on primary tumor	Molecular results on effusion specimens
1	Not detected	TP53 p.A161Pfs*8
2	TP53 p.E339*	TP53 p.E339* ESR1 p.G457V FBXW7 p.C466F PTEN p.Q17E
3	Not detected	Not detected
4	Not detected	KRAS p.G12C
5	TP53 p.C238*fs*1	TP53 p.C238*fs*1
6	Not detected	Not detected
7	MAP2K1 (MEK1) K57N	MAP2K1 p.K57N
8	EGFR p.L747_T751delinsP	EGFR p.L747_T751delinsP
9	EGFR amplification	EGFR amplification

### 2.6 Survival Analysis

**FIGURE 2:**
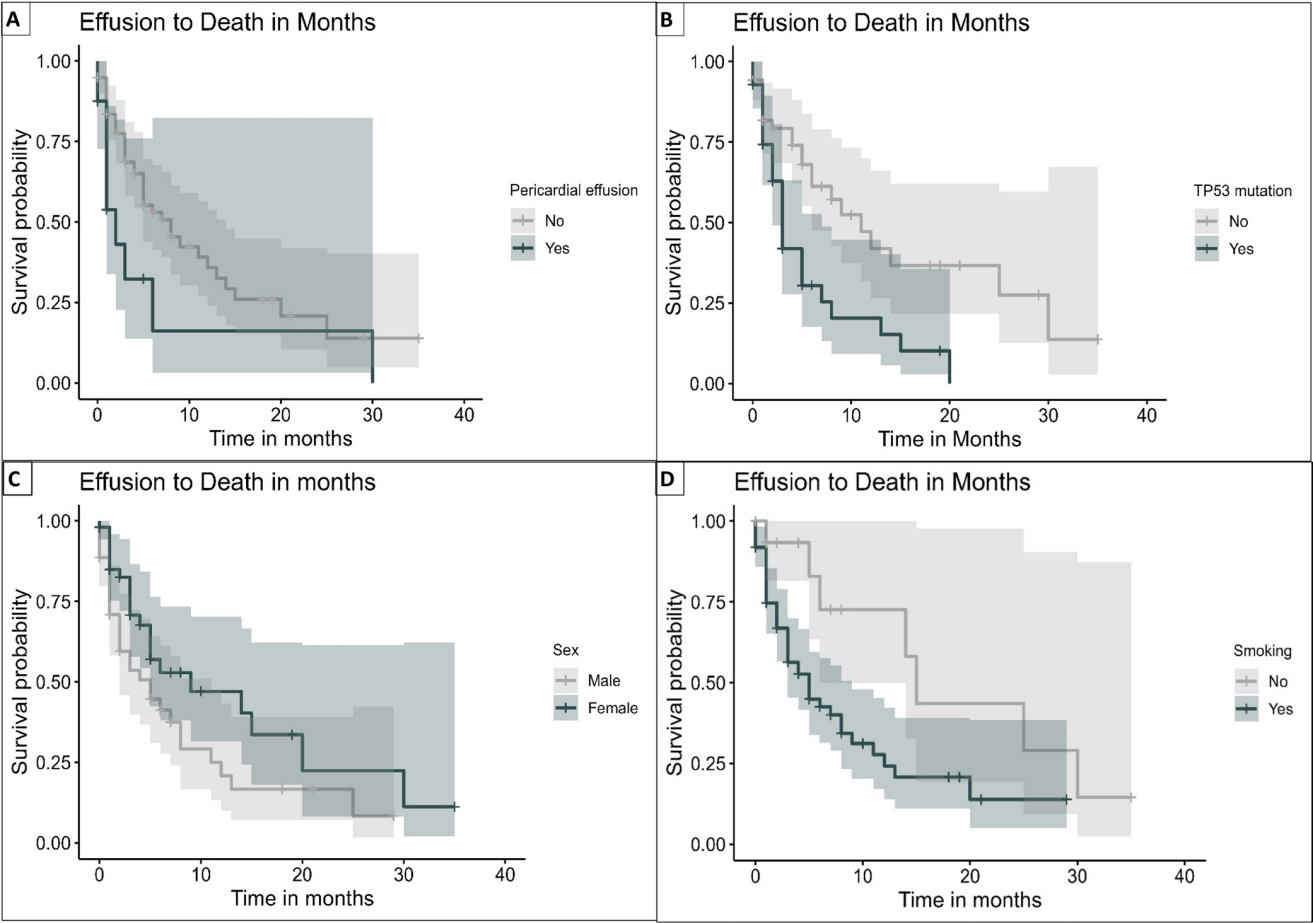
Survival analysis. Kaplan–Meier survival curves showing the association between the presence of concurrent pericardial effusion (3A), presence of TP53 mutation (3B), male sex (3C), and history of smoking (3D) and overall survival. [Color figure can be viewed at wileyonlinelibrary.com]

Overall, 51 of 94 patients (54%) died during the follow‐up period. Mortality was significantly higher in males than in females (56.9% vs. 43.1%, *p* = 0.033). Similarly, females had a decrease in risk of death compared to males (HR 0.56; 95% CI: 0.32, 0.99; *p* = 0.045). Death was significantly more common among patients with concurrent effusions than those without (52.9% vs. 47.1%, *p* = 0.002). Smokers had a 172% increased hazard of death compared to non‐smokers (HR: 2.72; 95% CI: 1.13, 6.52; *p* = 0.025). Median survival in non‐smokers compared with smokers was 15 months versus 5 months. Patients with pericardial effusions also exhibited an increase in hazard of death and shorter survival than those without (HR: 2.16; 95% CI: 1.09, 4.30; *p* = 0.027); the median survival being 2 months versus 8 months for those without associated pericardial effusion. There was no significant difference in survival across cellularity groups. Patients with TP53‐mutated effusions demonstrated higher mortality compared with TP53‐wild‐type cases (54.9% vs. 45.1%, *p* = 0.03). Moreover, survival analysis indicated an increase in hazard of death for those with TP53 mutation and median survival was markedly shorter in patients compared with those without (HR: 2.44; 95% CI: 1.37, 4.34; *p* = 0.002, median survival without mutation 11 months versus 3 months in those with TP53 mutation). FGFR1 mutations were less frequently observed among patients who died than among FGFR1‐positive survivors (11.8% vs. 88.2%, *p* = 0.03). No significant differences in mortality were noted with respect to other detected mutations (Figure 2).

## 3 Discussion

This study offers insights into the clinicopathological features, effusion‐specific clinical characteristics, cytological findings, and molecular profiles of patients with lung adenocarcinoma and MPE. The development of pleural effusion itself serves as an adverse prognostic marker in this population, reflecting more advanced disease and poor outcomes [1, 2, 3]. Integrating cytological and molecular parameters provides valuable diagnostic and prognostic information and may facilitate more refined patient stratification and management.

The minimum fluid volume and the required tumor cellularity for molecular analysis of effusion samples remain important but unresolved questions. Most published studies focus on cytological adequacy for diagnosis, with limited data addressing specific requirements for molecular testing. Beg et al. suggested that a minimum of 100 mL of serous fluid is generally sufficient for NGS, noting that nucleic acid yield and quality do not improve significantly beyond this volume [6]. They also highlighted that molecular testing can often be performed even in specimens with low tumor cellularity, due to the high sensitivity of contemporary molecular assays. However, their study did not define an optimal tumor cellularity threshold, and the cohort included a heterogeneous mix of primary malignancies, not limited to lung cancer. In a more recent study by Dalvi et al. among 36 effusion specimens subjected to molecular testing, the smallest fluid volume that allowed completion of their NGS panel was 20 mL, provided the T:NT ratio ranged between 20%–50% [10]. In our study, molecular analysis was performed with sample volumes as low as 5 mL. This sample had a T:NT ratio of 55%. Interestingly, samples with high tumor cellularity (> 50%) had a lower average volume (532 mL) compared to those with < 10% tumor cellularity (715 mL), although this difference was not statistically significant. Very low volume samples (≤ 10 mL) and low volume samples (11–50 mL) frequently demonstrated high tumor cellularity, with approximately two‐thirds of cases in each group exhibiting > 50% tumor content. In contrast, low tumor cellularity (< 10%) was predominantly observed in moderate to high volume effusion specimens (> 100 mL). Accurate assessment of tumor nuclei is particularly important in the background of inflammatory cells and reactive mesothelial cells. A specimen may appear overall cellular, yet true tumor cellularity must be distinguished from non‐neoplastic components when determining suitability for molecular analysis. When tumor cellularity is low, processing additional fluid for centrifugation may help concentrate tumor cells and increase the likelihood of successful testing. While both fluid volume and tumor cellularity are important considerations, tumor cellularity or T:NT fraction should be prioritized. In our study, even low‐volume specimens yielded successful molecular results when tumor cellularity was adequate. Although a flexible approach is practical, molecular testing decisions should be guided by tumor fraction on the cell block rather than fluid volume alone. Low‐volume specimens can still be tested if the T:NT ratio is high (laboratory‐dependent, but > 20% is generally acceptable). High‐volume specimens with low T:NT ratios, often due to admixture with reactive mesothelial or inflammatory cells, may benefit from additional centrifugation to concentrate tumor cells.

TTF‐1 expression was frequently observed in malignant effusions, underscoring its diagnostic utility in identifying pulmonary origin. TTF‐1 status was available in 93 of 94 cases (99%), with positivity noted in 82% of samples. Ma et al. reported a positivity rate of 68% in pericardial fluid specimens, which is consistent with the high prevalence seen in our study and makes it an indispensable biomarker for diagnosing lung adenocarcinoma, particularly in effusions [11, 12]. Beyond its diagnostic utility, TTF‐1 expression may provide additional clinical insight. Prior studies have demonstrated that TTF‐1 positivity is associated with improved clinical outcomes in patients with advanced lung adenocarcinoma treated with combined chemoimmunotherapy [13, 14]. However, no statistically significant difference in outcomes was observed between TTF‐1–positive and TTF‐1–negative effusions in lung adenocarcinoma in this study. Notably, patients with concurrent effusions more frequently exhibited TTF‐1 positivity compared with those without concurrent effusions. EGFR mutations were detected exclusively in TTF‐1 positive samples, aligning with previously published literature describing the association between TTF‐1 expression and EGFR‐mutant lung adenocarcinoma [15]. No significant correlations with other molecular alterations were identified in our analysis.

PD‐L1 testing on cytology cell blocks is well established and demonstrates good concordance with corresponding tissue biopsies [16]. PD‐L1 expression is widely used to predict responses to immune checkpoint inhibitors [9].

The reported prevalence of PD‐L1 positivity in lung adenocarcinoma generally ranges between 30% and 60%, depending on the study population and methodology. A recent large meta‐analysis of non–small cell lung cancer, including adenocarcinoma subtypes, demonstrated that approximately 55% of tumors exhibited PD‐L1 expression ≥ 1%, with 31% showing intermediate expression (1%–49%) and 23% demonstrating high expression (≥ 50%) [17, 18, 19, 20]. However, considerable variability exists across studies, influenced by factors such as the cutoff used to define positivity, the IHC clone and platform employed, as well as tumor histologic subtype and grade [19]. Importantly, the present study focuses specifically on malignant pleural effusion specimens from patients with lung adenocarcinoma, representing a clinically distinct and often more advanced disease population. PD‐L1 expression patterns in metastatic effusion samples may not fully mirror those observed in primary tumor resections or small biopsy specimens. Therefore, direct comparison of our findings with previously published cohorts should be interpreted with caution, given differences in patient selection, disease stage, specimen type, and methodological approaches.

We also observed a significant association between PD‐L1 positivity and the presence of loculated effusions. The observed association may reflect underlying immune dysregulation and chronic inflammation, as PD‐L1 positivity is indicative of an inflammatory tumor microenvironment [20]. Such an environment may promote fibrosis and septation formation, thereby predisposing patients to loculated effusions. Additionally, TTF‐1 positivity was significantly correlated with high PD‐L1 expression (> 50%), suggesting a potential interplay between tumor differentiation markers and immune checkpoint activity. These findings need to be further validated in a larger cohort.

With the advent of targeted therapies, the detection of actionable biomarkers in patients with malignant effusions has important clinical implications, with the potential to significantly influence disease management and patient outcomes [21, 22]. NGS of effusion specimens is increasingly relevant due to the ease of sample acquisition, their minimally invasive nature, and the feasibility of repeat testing [23]. The majority of patients (63/94, 67%) presented with concurrent effusions or effusions as the primary manifestation of disease. Although malignant effusions are generally associated with a poor prognosis, their presence provides a valuable opportunity for molecular evaluation using effusion specimens. The availability of effusion samples enabled several patients to receive targeted therapies based on actionable molecular findings. These findings underscore the clinical importance of cytology specimens and highlight the advantages of performing molecular testing on effusion samples. We performed molecular testing on low‐volume effusions with adequate tumor cellularity, supporting cytology‐based NGS as a primary diagnostic tool in clinical practice.

We identified seven actionable biomarkers in effusion specimens, including KRAS (29.7%), EGFR (15.9%), BRAF (8.5%), ALK fusions (4.2%), MET amplification (2.1%), ROS1 fusions (2.1%), and RET fusions (1%). Overall, TP53 was the most frequently detected mutation, present in 44.6% of cases. Ma et al. performed molecular analysis on pericardial fluid from 80 patients with non‐small cell lung cancer (70 adenocarcinomas) and similarly found TP53 to be the most common mutation (59%). Actionable mutations in their cohort included KRAS (34.1%), EGFR (15.9%), ALK fusions (4.5%), ROS1 fusions (2.3%), and RET fusions (2.3%), showing comparable frequencies to our pleural fluid specimens. Notably, the incidence of BRAF mutations was higher in our study (9% vs. 4.5%), whereas MET amplification was more frequent in their cohort (6.8% vs. 2%). Despite the high mortality in this population, 43 of 94 of our patients (46%) survived, of whom 27 (63%) received FDA‐approved targeted therapies based on biomarkers identified from effusion specimens. This underscores the clinical utility of molecular testing on effusions in guiding personalized treatment strategies. Notably, in one patient, NGS identified both lung cancer–associated mutations and an incidental JAK2 V617F mutation, suggesting the presence of a concurrent myeloproliferative neoplasm. This finding highlights the value of broad genomic profiling in detecting both expected tumor alterations and clinically significant, previously unrecognized conditions.

Molecular comparison between primary tumors and matched effusion specimens was available in limited patients, primarily because the majority of our cases 63/94 (67%) had presented concurrent pleural effusion and molecular testing was done only on the effusion specimen. Four primary tumors showed no detectable mutations; however, two of these had clinically relevant mutations (TP53 and KRAS) on subsequent effusion testing, while the remaining two remained mutation negative. Concordant molecular profiles between primary tumor and effusion specimens were observed in four patients. In one case, additional mutations (ESR1, FBXW7, and PTEN) were identified in the effusion specimen, with a shared TP53 mutation present in both the primary tumor and effusion specimen. The emergence of TP53 and KRAS mutations exclusively in effusion samples may reflect tumor evolution, clonal selection, or treatment‐related selective pressures occurring over the disease course. Detection of additional mutations (ESR1, FBXW7, PTEN) in one effusion sample suggests greater genomic complexity in advanced disease and shows that effusion testing can capture tumor heterogeneity. These findings require a larger cohort with available corresponding molecular analysis on primary versus effusion specimens.

The development of MPE in lung adenocarcinoma is a well‐recognized adverse prognostic indicator, with reported median survival ranging from 4 to 9 months [3]., Approximately half of our patients succumbed to their illness, with a median survival of 5.1 months, findings consistent with previously published data. As expected, male patients demonstrated poorer outcomes compared to females. Certain clinical features were also associated with worse prognosis, including a history of smoking and the presence of concurrent effusions, and when pleural effusion was accompanied by a pericardial effusion. Although higher tumor cellularity may suggest a more proliferative tumor burden, we did not observe a significant association between tumor cellularity and patient survival outcomes. TP53 mutations were strongly linked to poorer prognosis, aligning with their well‐established role as adverse molecular markers in cancers, irrespective of the presence of MPE. Interestingly, FGFR1 alterations were less frequent among patients who died. Aberrant FGFR pathway activation is reported in approximately 5.6%–8.8% of lung cancers and, while relatively uncommon, remains an area of active investigation due to its therapeutic potential [24]. Because its prognostic role is unclear, further study of FGFR1 mutations in MPE may clarify their impact on disease behavior and outcomes in this subgroup.

The limitations of this study include its retrospective design, which may restrict the generalizability of the findings. The NGS assay employed detects targeted, clinically relevant alterations such as single nucleotide variants (SNVs), small insertions and deletions (< 30 bp), whole‐gene copy number amplifications, and gene fusions within a predefined set of genes, but it cannot identify mutations below the assay's limit of detection or alterations in genes not covered by the panel.

To conclude, NGS testing was successful even in cytology samples with low cellularity, yielding clinically meaningful and actionable results that directly influenced patient management and outcomes. Distinct molecular and cytological patterns are noted in lung adenocarcinoma‐associated pleural effusions, with key prognostic implications which require larger studies to further validate. Male patients, smokers, those with associated pericardial effusions, and patients harboring TP53 mutations showed poorer outcomes following pleural effusion. Molecular profiling of effusion cytology specimens could even be considered a valuable primary diagnostic strategy, enabling timely identification of upfront actionable targets and facilitating precision‐guided therapy.

## Author Contributions

**Harpreet Virk:** drafting of manuscript, analysis of results, and review of slides. **Claire Michael and Omid Savari:** critical review of slides, results and manuscript. **Keri Ann Pfeil:** analysis of biostatistics. **Navid Sadri:** analysis of molecular data and revision of manuscript. **Aparna Harbhajanka:** study concept, design, review of slides, results and manuscript.

## Funding

The authors have nothing to report.

## Conflicts of Interest

The authors declare no conflicts of interest.

## Data Availability

The data that support the findings of this study are available from the corresponding author upon reasonable request.
